# Computed tomography to estimate cardiac preload and extravascular lung water. A retrospective analysis in critically ill patients

**DOI:** 10.1186/1757-7241-19-31

**Published:** 2011-05-23

**Authors:** Bernd Saugel, Konstantin Holzapfel, Jens Stollfuss, Tibor Schuster, Veit Phillip, Caroline Schultheiss, Roland M Schmid, Wolfgang Huber

**Affiliations:** 1 II. Medizinische Klinik und Poliklinik, Klinikum rechts der Isar der Technischen Universität München, Ismaninger Strasse 22, D-81675 München, Germany; 2 Institut für Röntgendiagnostik, Klinikum rechts der Isar der Technischen Universität München, Ismaninger Strasse 22, D-81675 München, Germany; 3 Radiologie, Klinikum Memmingen, Bismarck Strasse 23, D-87700 Memmingen, Germany; 4 Institut für Medizinische Statistik und Epidemiologie, Klinikum rechts der Isar der Technischen Universität München, Ismaninger Strasse 22, D-81675 München, Germany

## Abstract

**Background:**

In critically ill patients intravascular volume status and pulmonary edema need to be quantified as soon as possible. Many critically ill patients undergo a computed tomography (CT)-scan of the thorax after admission to the intensive care unit (ICU). This study investigates whether CT-based estimation of cardiac preload and pulmonary hydration can accurately assess volume status and can contribute to an early estimation of hemodynamics.

**Methods:**

Thirty medical ICU patients. Global end-diastolic volume index (GEDVI) and extravascular lung water index (EVLWI) were assessed using transpulmonary thermodilution (TPTD) serving as reference method (with established GEDVI/EVLWI normal values). Central venous pressure (CVP) was determined. CT-based estimation of GEDVI/EVLWI/CVP by two different radiologists (R1, R2) without analyzing software. Primary endpoint: predictive capabilities of CT-based estimation of GEDVI/EVLWI/CVP compared to TPTD and measured CVP. Secondary endpoint: interobserver correlation and agreement between R1 and R2.

**Results:**

Accuracy of CT-estimation of GEDVI (< 680, 680-800, > 800 mL/m^2^) was 33%(R1)/27%(R2). For R1 and R2 sensitivity for diagnosis of low GEDVI (< 680 mL/m^2^) was 0% (specificity 100%). Sensitivity for prediction of elevated GEDVI (> 800 mL/m^2^) was 86%(R1)/57%(R2) with a specificity of 57%(R1)/39%(R2) (positive predictive value 38%(R1)/22%(R2); negative predictive value 93%(R1)/75%(R2)). Estimated CT-GEDVI and TPTD-GEDVI were significantly different showing an overestimation of GEDVI by the radiologists (R1: mean difference ± standard error (SE): 191 ± 30 mL/m^2^, p < 0.001; R2: mean difference ± SE: 215 ± 37 mL/m^2^, p < 0.001). CT GEDVI and TPTD-GEDVI showed a very low Lin-concordance correlation coefficient (ccc) (R1: ccc = +0.20, 95% CI: +0.00 to +0.38, bias-correction factor (BCF) = 0.52; R2: ccc = -0.03, 95% CI: -0.19 to +0.12, BCF = 0.42). Accuracy of CT estimation in prediction of EVLWI (< 7, 7-10, > 10 mL/kg) was 30% for R1 and 40% for R2. CT-EVLWI and TPTD-EVLWI were significantly different (R1: mean difference ± SE: 3.3 ± 1.2 mL/kg, p = 0.013; R2: mean difference ± SE: 2.8 ± 1.1 mL/kg, p = 0.021). Again ccc was low with -0.02 (R1; 95% CI: -0.20 to +0.13, BCF = 0.44) and +0.14 (R2; 95% CI: -0.05 to +0.32, BCF = 0.53). GEDVI, EVLWI and CVP estimations of R1 and R2 showed a poor interobserver correlation (low ccc) and poor interobserver agreement (low kappa-values).

**Conclusions:**

CT-based estimation of GEDVI/EVLWI is not accurate for predicting cardiac preload and extravascular lung water in critically ill patients when compared to invasive TPTD-assessment of these variables.

## Background

In order to guide volume resuscitation adequately, early assessment of intravascular and pulmonary fluid status is a crucial goal in the management of critically ill patients in the emergency department or the intensive care unit (ICU).

However, assessment of the volume status using physical examination procedures is difficult and often inaccurate in these patients [1-5].

Portable chest radiography can be used for a rough estimation of intravascular volume status as well as lung water and pulmonary edema [6-8]. However, for monitoring small changes in lung water or for quantification of pulmonary edema chest roentgenograms are not accurate [7,8].

In ICU patients, invasive hemodynamic monitoring techniques are used for the assessment of hemodynamic variables. Transpulmonary thermodilution (TPTD) allows the measurement of cardiac preload (global end-diastolic volume index; GEDVI) and pulmonary fluid status (extravascular lung water index; EVLWI) [9-14].

In numerous studies the volumetric variable GEDVI has been shown to be accurate in the assessment of cardiac preload and volume responsiveness [9,15,16]. TPTD-based measurement of EVLWI has also been demonstrated to be accurate in animal studies compared to gravimetric measurements of extravascular lung water (EVLW) and in an autopsy study in humans compared to post-mortem lung weight [11,12,14]. In addition, there are data showing that EVLWI reflects severity of pulmonary disease and can predict outcome in patients with acute lung injury (ALI) or acute respiratory distress syndrome (ARDS) [17,18].

Nevertheless, determination of GEDVI and EVLWI using TPTD requires an arterial access, resulting in risks for complications and restricting these methods to the ICU [19].

In contrast, computed tomography (CT) has become a wide-spread diagnostic tool that is available even for non-ICU patients in many emergency departments. CT scanning of the thorax is very often performed due to basic clinical questions in the setting of critically ill patients in the first hours, frequently before establishing hemodynamic monitoring or admission to the ICU.

It has been shown that lung CT can help to understand the pathophysiology of ARDS and that it can influence clinical treatment decisions in ARDS patients [20-22]. One previous trial demonstrated that scoring systems based on CT lung morphology might help to identify patients with most severe forms of ARDS under study conditions [23].

Therefore, estimation of hemodynamic preload parameters and EVLWI using CT scans could potentially contribute to an early assessment of volume status, particularly in patients not (yet) under advanced hemodynamic monitoring.

The aim of our study was to investigate whether radiographic estimation of GEDVI, EVLWI and central venous pressure (CVP) using CT scanning of the thorax was able to contribute to an early, non-invasive estimation of hemodynamics in the clinical setting of critically ill patients. Radiographic estimation of GEDVI, EVLWI and CVP was compared to invasive assessment of these hemodynamic parameters using TPTD.

## Methods

### Patients

This was a retrospective analysis of a prospectively maintained TPTD database. We studied 30 critically ill patients treated in the medical ICU of a university hospital (Klinikum rechts der Isar, Technical University of Munich, Germany) who were examined by CT scanning of the thorax for clinical reasons unrelated to the study and who were monitored with TPTD using the PiCCO-System (Pulsion Medical Systems AG, Munich, Germany) at the same time. The study was approved by the local ethics committee.

### CT

30 CT scans (Siemens Volume-Zoom, Siemens Sensation, Siemens AG, Erlangen, Germany) of the 30 patients were independently analyzed by two experienced radiologists (radiologist 1 = R1 and radiologist 2 = R2). R1 and R2 were blinded to clinical findings and parameters determined by TPTD.

EVLWI was qualitatively estimated as elevated when engorged pulmonary vessels in the lung periphery exceeding the diameter of adjacent bronchi were seen. Thickening of bronchial walls secondary to excess fluid in the walls of the small airways (peribronchial cuffing), thickening of inter- and intralobular septae and ground-class opacities (i.e. areas of increased attenuation in the lung with preservation of bronchial and vascular markings) as features of interstitial pulmonary edema were considered indicative of moderately elevated EVLWI values (about 7-10 mL/kg). If consolidation of lung parenchyma (i.e. areas of increased attenuation in the lung with masking of bronchial and vascular markings accompanied by positive aerobronchogram) consistent with alveolar pulmonary edema was seen, EVLWI was classified as strongly elevated (> 10 mL/kg). In addition, for estimation of EVLWI density of lung parenchyma measured in the periphery of upper, lower and middle lobe was considered (radiographic attenuation values of normally aerated lung: -500 to -900 Hounsfield units (HU), poorly aerated lung: -100 to -500 HU, non-aerated lung: -100 to +100 HU) [24,25]. If larger areas of poorly and non-aerated lung were present, EVLWI was classified as strongly elevated (> 10 mL/kg). EVLWI was estimated according to the criteria mentioned above. Within the three categories (EVLWI < 7, 7 - 10, > 10 mL/kg) readers were asked to document a concrete value for EVLWI within the ranges mentioned based on subjective appreciation.

GEDVI was estimated by measuring the maximum short-axis diameter of right and left ventricle on axial images with diameters > 55 mm (left ventricle) and > 35 mm (right ventricle) indicating elevated preload. If the maximum of the short-axis diameter of the left ventricle was 55 - 60 mm and/or the maximum diameter of the right ventricle was 35 - 45 mm, GEDVI was classified as elevated (approximately > 800 mL/m^2^). When diameters of left and right ventricle exceeded 60 mm and 45 mm, respectively, GEDVI was classified as strongly elevated (approximately > 1000 mL/m^2^). In addition, the configuration of the inferior vena cava on the level of the hepatic veins was considered for the radiographic estimation of GEDVI with a biconvex configuration of the inferior vena cava indicative of elevated GEDVI [26]. The radiologists were asked to document a concrete value for GEDVI within three categories (GEDVI < 680, 680 - 800, > 800 mL/m^2^) based on subjective appreciation.

CVP values were quantitatively estimated by the radiologists based on subjective appreciation after evaluation of the filling of the inferior vena cava on the level of the hepatic veins [26].

In the clinical setting used in this trial, the average time for a radiologist to estimate EVLWI, GEDVI and CVP was about 5 minutes.

Twenty-eight of the 30 patients enrolled in this analysis received contrast medium (70 - 90 mL) for CT of the thorax.

### TPTD

GEDVI and EVLWI were measured in triplicate based on TPTD using a 5-French thermistor-tipped arterial line (Pulsiocath, Pulsion Medical Systems AG) inserted in the femoral artery and a commercially available hemodynamic monitor (PiCCO-Plus; PiCCO-2, Pulsion Medical Systems AG) as described before [5,27]. Global end-diastolic volume (GEDV) was indexed to the body surface area and EVLW was indexed to the predicted body weight. In the patients included in the retrospective analysis, TPTD had been performed within a mean of 2.25 hours before or after the CT scan.

### Endpoints

The primary endpoints were the diagnostic accuracy, sensitivity, specificity, positive predictive value (PPV) and negative predictive value (NPV) of radiologically estimated GEDVI and EVLWI regarding elevated and decreased values compared to TPTD-derived GEDVI and EVLWI.

The secondary endpoints were the interobserver correlation and agreement between the two radiologists and the analysis of radiologically estimated CVP compared to measured CVP and comparison of these parameters to GEDVI and EVLWI.

### Statistical analysis

Diagnostic accuracy, sensitivity, specificity, PPV and NPV were calculated with corresponding 95% confidence intervals (95% CI). The Spearman correlation coefficient (rho) was used to investigate bivariate correlations of quantitative measurements. Paired t-test was used to assess systematic differences in competitive measurements. To illustrate agreement of interesting measurements Bland-Altman figures and scatter plots with optimal reference line (45 degree) are provided [28]. The concordance correlation coefficient proposed by Lin (ccc) was used to evaluate agreement of quantitative measurements in consideration of accuracy and precision [29]. In this term the bias correction factor (BCF) was reported which measures how far the best-fit line deviates from the optimal line at 45 degrees (perfect agreement). No deviation from the 45 degree line occurs when BCF = 1 (possible range of values > 0 to 1). Per definition Lin's ccc is determined by the product of Pearson correlation coefficient (r) and the BCF (ccc = r*BCF), thus both - information of systematical deviation and correlation of two measurements - is combined in one index, which takes values from -1 to 1. Statistical analysis was performed using PASW Statistics (version 17; SPSS inc., Chicago, Illinois, USA) and the statistical software package R version 2.7.1 (R Foundation for Statistical Computing, Vienna, Austria). All tests were conducted two-sided and statistical significance was considered at p < 0.05.

## Results

### Patients and patients' characteristics

Thirty critically ill ICU patients were enrolled in this study. The patients' basic demographic data and clinical characteristics including reason for ICU admission, ICU treatment, laboratory tests, and ICU outcome are presented in Table 1.

**Table 1:** Patients' demographic data, patients' clinical characteristics, and reason for intensive care unit admission

Basic demographic data	
Sex (female/male)	14/16

Age, years	66 (27 - 83)

Height, cm	170 (150 - 180)

Weight, kg	68 (44 - 112)

**Patients' clinical characteristics on the day of enrollment in the study**	

Simplified Acute Physiology Score (SAPS-II)	50 (22 - 63)

Therapeutic Intervention Scoring System (TISS-score)	22 (10 - 35)

Serum creatinine, mg/dL	1.8 (0.5 - 4.6)

Blood urea nitrogen, mg/dL	52 (8 - 106)

Serum bilirubin, mg/dL	2.7 (0.3 - 23.2)

Aspartate aminotransferase, U/L	122 (11 - 4977)

Leukocyte count, G/L	16.2 (0.1 - 50.0)

C-reactive proteine, mg/dL	7.9 (0.1 - 45.7)

Hematocrit level, %	29 (23 - 47)

Hemoglobin, g/dL	9.9 (7.3 - 16.0)

Heart rate, beats per minute	94 (57 - 144)

Need for catecholamine therapy, n (%)	23 (77%)

Norepinephrine dose, μg/kg/min	0.16 (0 - 1.89)

Need for mechanical ventilation, n (%)	25 (83%)

Positive end-expiratory pressure, cmH_2_O	8 (4 - 16)

Peak pressure, cmH_2_O	24 (13 - 31)

Mean airway pressure, cmH_2_O	14 (7 - 21)

Fraction of inspired oxygen	0.5 (0.3 - 1.0)

Tidal volume, mL	500 (300 - 842)

pH	7.33 (7.18 - 7.60)

Arterial partial pressure of carbon dioxide, mmHg	37.0 (21.0 - 64.5)

Arterial partial pressure of oxygen, mmHg	89.1 (58.0 - 160.0)

Bicarbonate, mEq/L	20.9 (11.4 - 36.4)

Base excess, mEq/L	-3.9 (-14.1 - 11.7)

**Reason for ICU admission**	

Sepsis with multiple organ dysfunction syndrome, n (%)	9 (30%)

Pneumonia and acute respiratory insufficiency, n (%)	8 (27%)

Cirrhosis of the liver, n (%)	4 (13%)

Pancreatitis, n (%)	3 (10%)

Cardiac arrest with need for cardiopulmonary resuscitation, n (%)	2 (7%)

Gastrointestinal bleeding, n (%)	2 (7%)

Renal failure, n (%)	1 (3%)

Pulmonary embolism, n (%)	1 (3%)

**Outcome**	

Intensive care unit mortality, n (%)	17 (57%)

Data are presented as median (range) where applicable. ICU, intensive care unit.

### TPTD results

At the time of enrollment, mean TPTD-derived GEDVI was 685 ± 154 mL/m^2 ^(range: 412 to 1044 mL/m^2^), mean TPTD-derived EVLWI was 11.6 ± 6.4 mL/kg (range: 4 to 38 mL/kg), and mean measured CVP was 15.9 ± 6.3 mmHg (range: 4 to 32 mmHg). The distribution of GEDVI, EVLWI, and CVP values categorized according to the used thresholds is presented in Table 2.

**Table 2:** Transpulmonary thermodilution-derived hemodynamic variables and measured central venous pressure

TPTD-derived GEDVI		
GEDVI < 680 mL/m^2^, n (%)	GEDVI 680 - 800 mL/m^2^, n (%)	GEDVI > 800 mL/m^2^, n (%)

14 (47%)	9 (30%)	7 (23%)

**TPTD-derived EVLWI**		

EVLWI < 7 mL/kg, n (%)	EVLWI = 7 - 10 mL/kg, n (%)	EVLWI > 10 mL/kg, n (%)

5 (17%)	11 (37%)	14 (47%)

**CVP (measured)**		

CVP < or = 9 mmHg	CVP > 9 mmHg	

5 (17%)	25 (83%)	

Distribution of transpulmonary thermodilution (TPTD)-derived values of global end-diastolic volume index (GEDVI) and extravascular lung water index (EVLWI) as well as values of measured central venous pressure (CVP) according to the used thresholds. Data are presented as absolute numbers (n) with percentages in parentheses.

### CT-scan results

Estimation of GEDVI based on CT resulted in a mean estimated GEDVI of 877 ± 137 mL/m^2 ^(range: 700 to 1100 mL/m^2^) for R1 and 900 ± 117 mL/m^2 ^(range: 750 to 1100 mL/m^2^) for R2. Mean radiologically estimated EVLWI was 8.3 ± 1.9 mL/kg (range: 5 to 12 mL/kg) (R1) and 8.9 ± 2.2 mL/kg (range: 5 to 14 mL/kg) (R2). Mean CVP estimated by R1 and R2 was 8.6 ± 2.3 mmHg (range: 5 to 12 mmHg) and 8.2 ± 2.2 mmHg (range: 5 to 14 mmHg), respectively. In Table 3 the distribution of estimated GEDVI, EVLWI, and CVP values categorized according to the used thresholds is shown.

**Table 3:** Computed tomography-based estimation of hemodynamic parameters

CT-based estimation of hemodynamic variables			
	**GEDVI (estimated)**		

	GEDVI < 680 mL/m^2^, n (%)	GEDVI 680 - 800 mL/m^2^, n (%)	GEDVI > 800 mL/m^2^, n (%)

**R1**	0 (0%)	14 (47%)	16 (53%)

**R2**	0 (0%)	12 (40%)	18 (60%)

	**EVLWI (estimated)**		

	EVLWI < 7 mL/kg, n (%)	EVLWI = 7 - 10 mL/kg, n (%)	EVLWI > 10 mL/kg, n (%)

**R1**	4 (13%)	21 (70%)	5 (17%)

**R2**	4 (13%)	19 (63%)	7 (23%)

	**CVP (estimated)**		

	CVP < or = 9 mmHg	CVP > 9 mmHg	

**R1**	20 (67%)	10 (33%)	

**R2**	22 (73%)	8 (27%)	

Distribution of radiographically estimated values of global end-diastolic volume index (GEDVI), extravascular lung water index (EVLWI), and central venous pressure (CVP) according to the used thresholds. Data are presented as absolute numbers (n) with percentages in parentheses. CT, computed tomography; R1, radiologist 1; R2, radiologist 2.

### Comparison of CT-based estimations of the two radiologists (R1 and R2)

Comparison of the two radiologists' estimations of GEDVI, EVLWI and CVP without any categorization and determination of the interobserver correlation showed a low ccc for all three variables (GEDVI: ccc = +0.64, 95% CI: +0.38 to +0.81, BCF of 0.97; EVLWI: ccc = +0.63, 95% CI: +0.37 to +0.80, BCF of 0.96; CVP: ccc = +0.63, 95% CI: +0.36 to +0.80, BCF of 0.99). After categorization of the radiologists' estimations of GEDVI, EVLWI and CVP (GEDVI < 680, 680 - 800, > 800 mL/m^2^; EVLWI < 7 or >/= 7 mL/kg; CVP 1-9 or > 9 mmHg) the interobserver agreement showed poor kappa-values (GEDVI: kappa = 0.46; EVLWI: kappa = 0.71; CVP: kappa = 0.53).

### Comparison of TPTD-GEDVI vs. GEDVI estimated using CT scan

**Figure 1:**
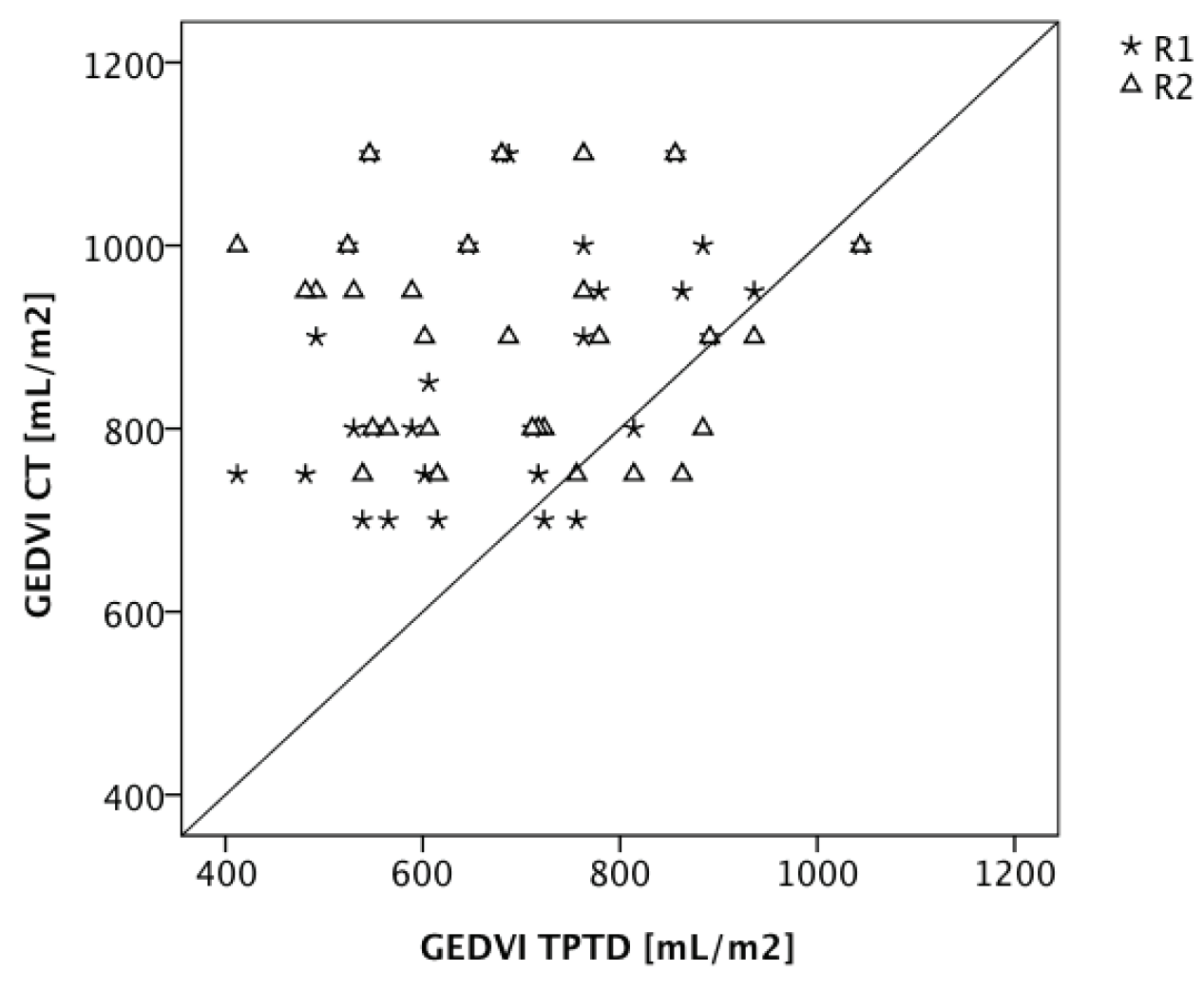
**CT-based GEDVI estimation compared to TPTD-derived GEDVI**. Scatter plot showing GEDVI values derived from TPTD (GEDVI TPTD) compared to GEDVI estimations based on CT scans (GEDVI CT) by radiologist 1 (R1) and radiologist 2 (R2).

**Figure 2:**
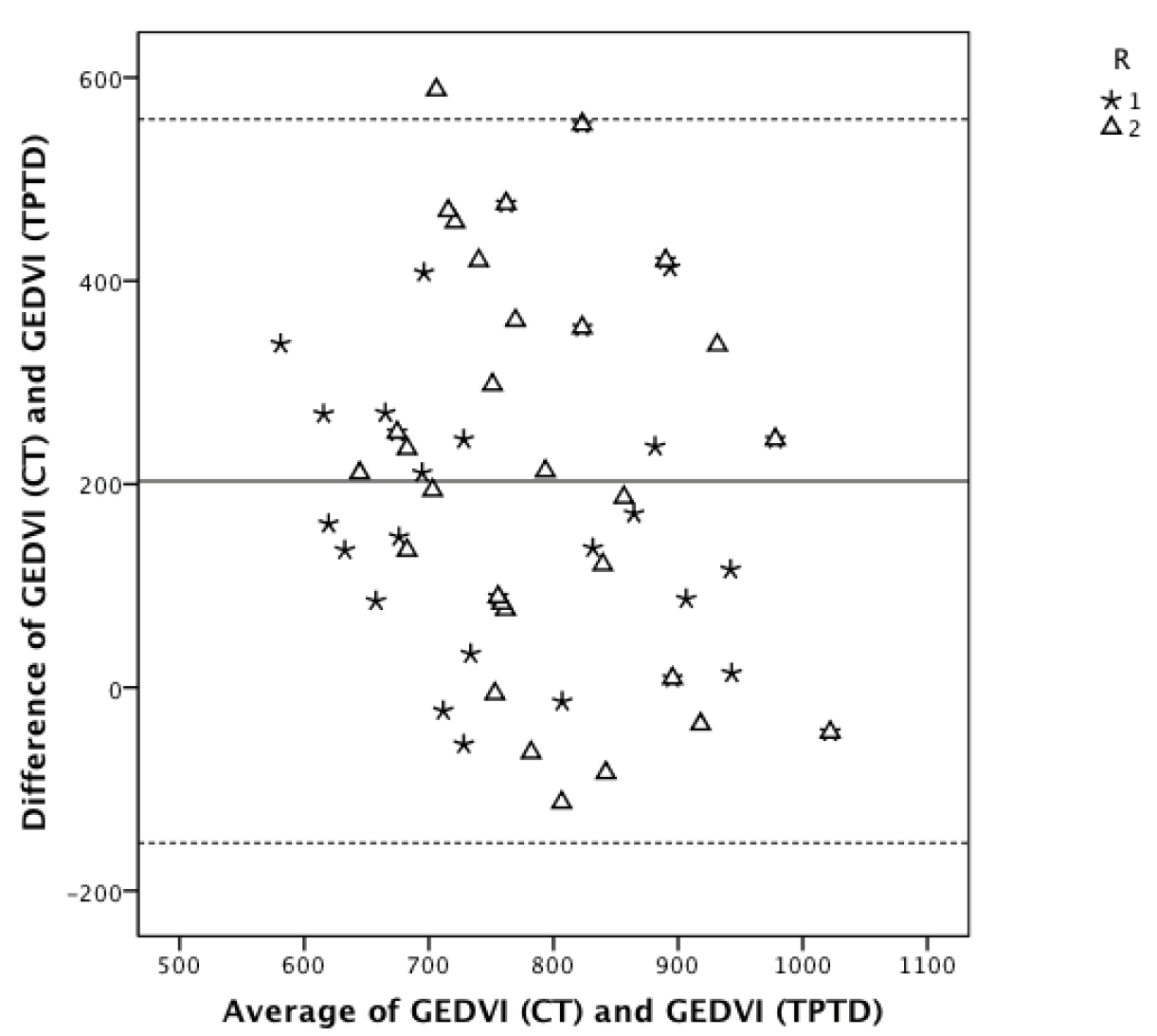
**CT-based GEDVI estimation compared to TPTD-derived GEDVI**. **Bland-Altman analysis**. Bland-Altman figure showing individual agreement between radiographic estimation of GEDVI (GEDVI (CT)) and TPTD measurement of GEDVI (GEDVI (TPTD)). R1, radiologist 1; R2, radiologist 2. The middle line indicates the mean difference between variables determined using TPTD and radiographic estimation. The upper and lower dashed lines indicate the 95% limits of agreement (mean difference ± 1.96*SD).

GEDVI values estimated by the radiologists and TPTD-derived GEDVI values were significantly different (R1: mean difference ± standard error (SE): 191 ± 30 mL/m^2^, p < 0.001; R2: mean difference ± SE: 215 ± 37 mL/m^2^, p < 0.001) with an overestimation of radiographic estimated GEDVI values in 90% of false estimations (Figure 1). Comparison of GEDVI values estimated using CT and TPTD-derived GEDVI values showed a very low ccc (R1: ccc = +0.20, 95% CI: +0.00 to +0.38; R2: ccc = -0.03, 95% CI: -0.19 to +0.12) with a BCF of 0.52 (R1) and 0.42 (R2). To evaluate the individual agreement between radiographic estimations of GEDVI and TPTD assessment of GEDVI, a Bland-Altman figure is presented in Figure 2. Diagnostic accuracy of radiographic estimation of GEDVI (after categorization of GEDVI in 3 categories: GEDVI < 680, 680 - 800, > 800 mL/m^2^) using CT of the thorax was 33% (R1; 95% CI: 17% to 53%) and 27% (R2; 95% CI: 12% to 46%). Despite a number of markedly decreased TPTD-GEDVI measurements, none of the radiologists classified any GEDVI value as decreased. Table 4 shows predictive capabilities of CT-based GEDVI estimation with regard to GEDVI derived from TPTD.

**Table 4:** Predictive capabilities of computed tomography-based estimation of global end-diastolic volume index

		CT-based estimation of GEDVI vs. TPTD-derived GEDVI		
		
		**GEDVI < 680 mL/m**^**2**^	**GEDVI = 680 - 800 mL/m**^**2**^	**GEDVI > 800 mL/m**^**2**^
**Radiologist 1**	Sensitivity	0 (0 to 23)	44 (14 to 79)	86 (42 to 99)

	Specificity	100 (79 to 100)	52 (30 to 74)	57 (34 to 77)

	PPV	not estimable due to zero counts in numerator	29 (8 to 58)	38 (15 to 65)

	NPV	53 (34 to 72)	69 (41 to 89)	93 (66 to 99)

**Radiologist 2**	Sensitivity	0 (0 to 23)	44 (14 to 79)	57 (18 to 90)

	Specificity	100 (79 to 100)	62 (38 to 82)	39 (20 to 61)

	PPV	not estimable due to zero counts in numerator	33 (10 to 65)	22 (6 to 48)

	NPV	53 (34 to 72)	72 (47 to 90)	75 (43 to 95)

Sensitivity, specificity, positive predictive value (PPV), negative predictive value (NPV) given as percentages with 95% confidence intervals (95% CI) in parentheses for computed tomography (CT)-based estimations of global end-diastolic volume index (GEDVI) with regard to GEDVI derived from transpulmonary thermodilution (TPTD) are shown separately for radiologist 1 and radiologist 2.

### Comparison of TPTD-EVLWI vs. EVLWI estimation based on CT scan

**Figure 3:**
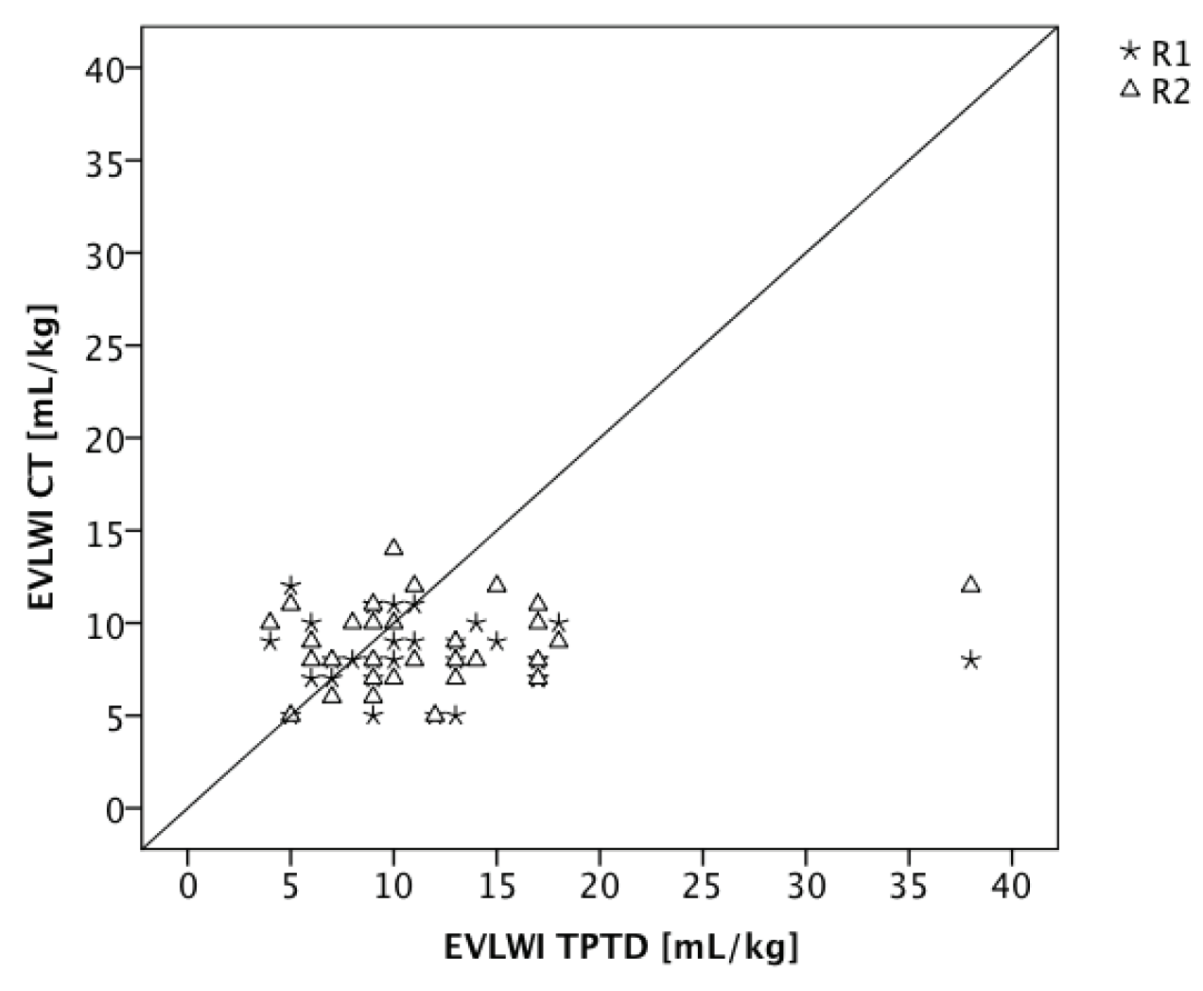
**CT-based EVLWI estimation compared to TPTD-derived EVLWI**. Scatter plot showing EVLWI values determined by TPTD (EVLWI TPTD) compared to EVLWI estimation based on CT scans (EVLWI CT) by radiologist 1 (R1) and radiologist 2 (R2).

**Figure 4:**
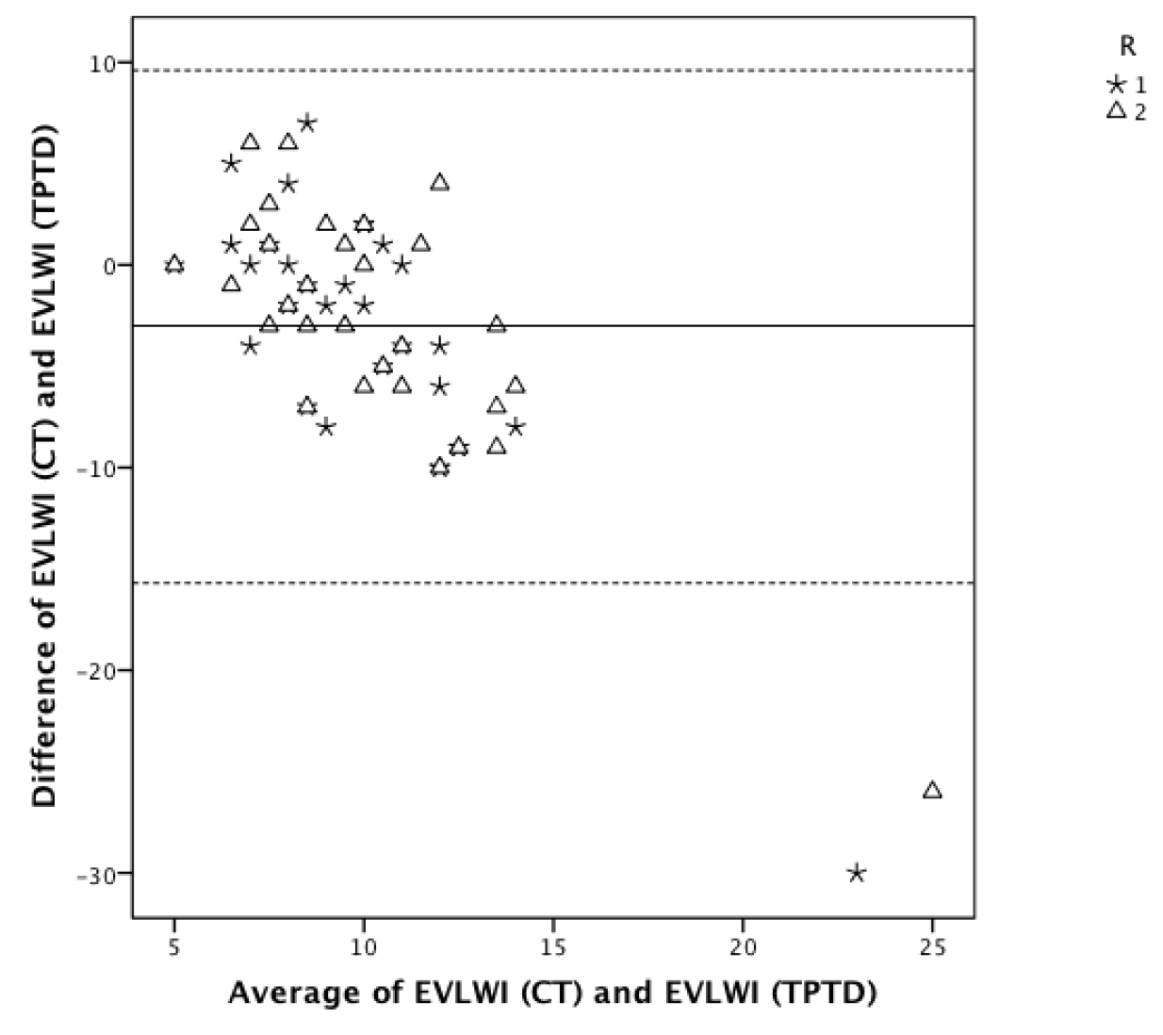
**CT-based EVLWI estimation compared to TPTD-derived EVLWI**. **Bland-Altman analysis**. Bland-Altman figure showing individual agreement between radiographic estimation of EVLWI (EVLWI (CT)) and TPTD measurement of EVLWI (EVLWI (TPTD)). R1, radiologist 1; R2, radiologist 2. The middle line indicates the mean difference between variables determined using TPTD and radiographic estimation. The upper and lower dashed lines indicate the 95% limits of agreement (mean difference ± 1.96*SD).

Radiographic estimation of EVLWI according to the used thresholds (EVLWI < 7, 7 - 10, > 10 mL/kg) showed a diagnostic accuracy of 30% (R1; 95% CI: 14% to 46%) and 40% (R2; 95% CI: 22% to 58%), respectively. Sensitivity, specificity, PPV, and NPV for CT-based estimations of EVLWI for R1 and R2 are shown in Table 5. EVLWI estimated using CT and TPTD-derived EVLWI were significantly different (R1: mean difference ± SE: 3.3 ± 1.2 mL/kg, p = 0.013; R2: mean difference ± SE: 2.8 ± 1.1 mL/kg, p = 0.021) (Figure 3). ccc was low with -0.02 (R1; 95% CI: -0.20 to +0.13, BCF of 0.44) and +0.14 (R2; 95% CI: -0.05 to +0.32, BCF of 0.53). The corresponding Bland-Altman figure is presented in Figure 4.

**Table 5:** Predictive capabilities of computed tomography-based estimation of extravascular lung water index

		CT-based estimation of EVLWI vs. TPTD-derived EVLWI		
		
		EVLWI < 7 mL/kg	EVLWI 7 - 10 mL/kg	EVLWI > 10 mL/kg
**Radiologist 1**	Sensitivity	20 (0.5 to 72)	64 (31 to 89)	7 (0.1 to 34)

	Specificity	88 (69 to 97)	26 (9 to 51)	75 (48 to 93)

	PPV	25 (0.6 to 81)	33 (15 to 57)	20 (0.5 to 72)

	NPV	85 (65 to 96)	56 (21 to 86)	48 (28 to 69)

**Radiologist 2**	Sensitivity	20 (0.5 to 72)	64 (31 to 89)	29 (8 to 58)

	Specificity	88 (69 to 97)	37 (16 to 62)	81 (54 to 96)

	PPV	25 (0.6 to 81)	37 (16 to 62)	57 (18 to 90)

	NPV	85 (65 to 96)	64 (31 to 89)	57 (34 to 77)

Sensitivity, specificity, positive predictive value (PPV), negative predictive value (NPV) given as percentages with 95% confidence intervals (95% CI) in parentheses for computed tomography (CT)-based estimations of extravascular lung water index (EVLWI) with regard to EVLWI derived from transpulmonary thermodilution (TPTD) are shown separately for radiologist 1 and radiologist 2.

### Comparison of CVP vs. radiographic CVP estimation

The prediction of CVP (CVP 1 - 9 or > 9 mmHg) estimated using CT showed a diagnostic accuracy of only 43% for both radiologists. Sensitivity for prediction of elevated CVP (CVP > 9 mmHg) was only 36% (R1) and 32% (R2) with a specificity of 80% (R1) and 100% (R2) (table 6). PPV for prediction of elevated CVP was 90% (R1) and 100% (R2). NPV was 20% (R1) and 23% (R2). CVP estimated by the radiologist using CT and assessed CVP were significantly different (R1: mean difference ± SE: 7.3 ± 1.1 mmHg, p < 0.001; R2: mean difference ± SE: 7.6 ± 1.1 mmHg, p < 0.001). ccc was again low with +0.08 (R1; 95% CI: -0.03 to +0.19, BCF of 0.29) and +0.11 (R2; 95% CI: -0.01 to +0.21, BCF of 0.27).

**Table 6:** Predictive capabilities of computed tomography-based estimation of central venous pressure

	CVP CT (R1) vs. CVP	95% CI lower	95% CI upper		CVP CT (R2) vs. CVP	95% CI lower	95% CI upper
Sensitivity	36%	17%	55%	Sensitivity	32%	14%	50%

Specificity	80%	45%	> 99%	Specificity	100%	49%	100%

PPV	90%	71%	> 99%	PPV	100%	63%	100%

NPV	20%	3%	38%	NPV	23%	5%	40%

Sensitivity, specificity, positive predictive value (PPV), negative predictive value (NPV) and 95% confidence intervals (95% CI) for computed tomography (CT)-based estimations of central venous pressure (CVP CT) with regard to measured elevation of central venous pressure (CVP; CVP > 9 mmHg) are shown. CVP CT is separately shown for radiologist 1 (R1) and radiologist 2 (R2).

### Comparison CVP vs. GEDVI in prediction of volume status

Measured CVP was analyzed with regard to measured GEDVI. For predicting TPTD-derived volume status (GEDVI < 680, 680 - 800, > 800 mL/m^2^) the assessment of CVP (CVP < 1, 1 - 9, > 9 mmHg) showed a diagnostic accuracy of 27% with a NPV for hypovolemic fluid status (GEDVI < 680 mL/m^2^) of 53% (sensitivity 0%, specificity 100%, PPV 0%). CVP values and GEDVI values did not significantly correlate (Spearman's correlation coefficient rho = -0.143, p = 0.45).

In addition CVP did not significantly correlate to EVLWI values assessed by TPTD (Spearman's correlation coefficient rho = +0.222, p = 0.24). The CVP showed a diagnostic accuracy in estimation of EVLWI of 83%. Sensitivity for prediction of pulmonary edema/fluid overload (EVLWI >/= 7 ml/kg; CVP > 9 mmHg) was 88% (specificity 40%, PPV 88%, NPV 40%).

## Discussion

CT scans of the thorax are frequently performed in critically ill patients during the first hours in the emergency department or after ICU admission even before hemodynamic monitoring can be established. Therefore, using routine CT scans, CT-based estimation of preload and pulmonary fluid status might have considerable impact on early volume resuscitation in critically ill patients.

This study investigated whether radiographic estimation (by two independent radiologists) of GEDVI, EVLWI and CVP using CT scans of the thorax is able to evaluate intravascular and pulmonary fluid status in critically ill patients. To obtain representative data in a *clinical routine setting*, we did not use analyzing software, and the radiologists were totally blinded to clinical, laboratory and TPTD-derived information. The main results of this study can be summarized as follows: In critically ill patients estimation of hemodynamic parameters (GEDVI, EVLWI) or CVP using CT is not accurate when compared to invasive assessment of these variables using TPTD or CVP measurement, respectively. TPTD-derived values for GEDVI and EVLWI were significantly different from GEDVI and EVLWI values estimated using CT. Estimation of GEDVI is not satisfactorily accurate, sensitive or specific for prediction of a *hypovolemic *volume status (defined by TPTD, GEDVI < 680 mL/m^2^). Regarding prediction of *hypervolemia *(GEDVI > 800 mL/m^2^) radiographic estimation showed slightly better predictive capabilities with low PPVs. For predicting EVLWI and CVP the radiographic estimation is not sufficiently accurate, sensitive or specific.

These results are partially in contrast to previous studies.

A recently published animal study by Kuzkov et al. found an association of lung tissue volume assessed by quantitative CT and EVLWI (determined by TPTD and postmortem gravimetry) in 7 sheep with ALI [30]. However, it has been demonstrated that EVLWI data obtained in animal models are not easily transferable to humans because a species-specific correction factor might be needed for the calculation of EVLWI [31,32]. Correspondingly, another animal study in dogs found that EVLWI values markedly increased when ALI was induced whereas lung tissue density assessed by CT did not alter [33].

In contrast to the results of our clinical study, there are data from an in-vitro study using a lung specimen suggesting that accurate assessment of lung water can be achieved by CT scanning using analyzing software under study conditions [34]. However, these results are hardly transferable to critically ill patients, because the study was conducted using special analyzing software and a lung model (air-dried, ex-sanguine human lung).

In a case-series of patients with ARDS quantification of lung edema by computed tomography using dedicated analyzing software showed a good correlation with measurements of lung edema using the thermal indocyanine green-dye double-dilution method [35]. However this study performed in an experimental setting was restricted to 14 patients and used experimental analyzing software for CT-based estimation of EVLW, which is not routinely available and therefore does not reflect standard clinical conditions. By contrast, our protocol was deliberately aimed at routine standard conditions and the radiologists read the CT scans in a clinical setting without the support of quantitative CT analyzing software. In contrast to previous studies, the predictive capabilities of radiographic estimation of hemodynamic parameters observed in the present study are therefore applicable to a realistic clinical routine setting.

In our study the investigating radiologists were completely blinded to the clinical and laboratory data of the patients in order to exclude suggestive data related to the pre-existing hemodynamic status. This might have impaired the radiological estimation when compared to clinical routine.

In the present trial CT-based estimation of CVP was not sufficiently accurate, sensitive or specific. However, regarding the use of CVP values for the assessment of cardiac preload there is increasing evidence that several factors can influence CVP determination in critically ill patients and that CVP is therefore not able to reflect cardiac preload and predict volume responsiveness [9,36]. For example, CVP can be overestimated in patients with increased intraabdominal pressure or mechanical ventilation with high positive end-expiratory pressure [37].

One might argue that cardiac volume and pulmonary vascular status might have been affected by the fast intravenous injection of about 70 - 90 mL of contrast-medium potentially resulting in an overestimation of cardio-pulmonary filling. However, 12 of the 30 TPTD measurements were performed before the application of contrast-medium, thus excluding a bias by contrast injection.

Finally, failure of CT-based estimation to exactly predict hemodynamic parameters does not necessarily mean that CT can not provide important data improving the interpretation of hemodynamic measurements. For example, CT might be useful in the interpretation of elevated EVLWI resulting from inflammation or cardiac congestion. Furthermore, interdisciplinary training of the radiologists and further development of diagnostic algorithms might improve radiological assessment of volume status. However, performing CT in critically ill patients is associated with potential risks since CT requires patient transport and is associated with X-ray exposure.

TPTD was used as the reference method for assessment of cardiac preload and EVLWI in the present study. It is important to emphasize that this advanced and invasive hemodynamic monitoring technique has some inherent limitations and can not be considered as an absolute gold standard for determination of a patient's volume status: Since an arterial catheter and a central venous catheter is required to perform TPTD measurements, this method is usually restricted to ICUs and is not available for emergency department or normal ward patients. Although there are data from previous studies that TPTD-derived volumetric parameters of cardiac preload might predict volume responsiveness more accurately than CVP or pulmonary artery wedge pressure (obtained using a pulmonary artery catheter), in certain patients with cardiovascular disorders (e.g. intracardiac left-right-shunt, valvulopathies, aortic aneurysms) the TPTD-based determination of cardiac preload (GEDVI) can be adulterated [9,10,38,39]. In addition, the recommended and established thresholds for normal values of hemodynamic variables derived from TPTD were defined based on data from studies in selected populations of patients and might therefore not be unrestrictedly applicable for all patients. Results from an autopsy study recently confirmed the recommended normal value of EVLWI defined by the manufacturer of the device [14]. Regarding GEDVI, there is evidence from one trial that normal values of this preload parameter should be adjusted to sex and age in neurosurgery patients [40]. In addition, a recent study in medical ICU patients suggested that GEDVI might be corrected for cardiac ejection fraction for better prediction of preload [41].

### Limitations of the study

- In the present study we compared radiographic CT-based estimation of hemodynamic variables to invasively assessed hemodynamic parameters using TPTD. Although TPTD is established for assessment of cardiac preload and pulmonary hydration, this technique has some inherent limitations and can therefore not be considered the absolute gold standard method for determination of hemodynamics.

- This monocentric study was conducted retrospectively in a medical ICU and the results are therefore not generalizable to other patient populations. The findings of this pilot study rather need to be confirmed in a prospective trial in a larger number of patients.

- Another limitation of this retrospective data analysis is that there was a time interval of a mean of 2 hours between TPTD and the CT scan. In a future prospective study TPTD should be performed directly before and after CT.

## Conclusions

The results of our study suggest that estimation of GEDVI and EVLWI using standard CT scans of the thorax is not accurate in critically ill patients in a clinical setting without the support of quantitative CT analyzing software when compared to invasive assessment of these variables using TPTD. At this point CT-based estimation can not provide reliable and reproducible quantification of fluid overload, low cardiac preload or pulmonary edema defined by the TPTD variables GEDVI and EVLWI and therefore seems to be of limited use for early assessment of volume status in critically ill patients. However, it should be mentioned, that prognostic capabilities of radiographic estimation can probably be improved by interdisciplinary training and more detailed clinical information provided to the radiologist as well as improved diagnostic algorithms. An intriguing approach in further prospective trials in a larger number of patients could be to develop an objective formula for CT-based estimations of GEDVI and EVLWI.

## Abbreviations

ALI: acute lung injury; ARDS: acute respiratory distress syndrome; BCF: bias correction factor; ccc: concordance correlation coefficient proposed by Lin; CT: computed tomography; CVP: central venous pressure; EVLW: extravascular lung water; EVLWI: extravascular lung water index; GEDV: global end-diastolic volume; GEDVI: global end-diastolic volume index; HU: Hounsfield units; ICU: intensive care unit; NPV: negative predictive value; PPV: positive predictive value; R1: radiologist 1; R2: radiologist 2; SE: standard error; TPTD: transpulmonary thermodilution; 95% CI: 95% confidence interval.

## Competing interests

There is no financial support for the research to disclose. WH is member of the Medical Advisory Board of Pulsion Medical Systems AG. All other authors have no conflict of interest to declare.

## Authors' contributions

BS, VP, CS and WH contributed to the conception and design of the study. They were responsible for acquisition, analysis and interpretation of data. BS and WH drafted the manuscript. RMS participated in its design and coordination and helped to draft the manuscript. KH and JS are experienced radiologists. They both participated in the design of the study and read the CT scans. TS participated in the design of the study and performed the statistical analysis. All authors read and approved the final manuscript.
